# Interaction of Mannitol and Sucrose with Gellan Gum in Freeze-Dried Gel Systems

**DOI:** 10.1007/s11483-018-9536-5

**Published:** 2018-05-22

**Authors:** Mattia Cassanelli, Ian Norton, Tom Mills

**Affiliations:** School of Chemical Engineering, University of Birmingham, Edgbaston, Birmingham, B15 2TT UK

**Keywords:** Gellan gum, Freeze drying, Microstructure, Sucrose, Mannitol

## Abstract

The effect of sucrose and mannitol addition to low-acyl (LA) gellan gum gels at both the molecular and macroscopic levels prior to, and after freeze-drying has been investigated. It has been shown that the gel network order as well as the mechanical properties are changed with the solute content, especially in the case of sucrose. The freeze-dried gel structure, containing either mannitol or sucrose, was studied, reporting for the first time the interaction of mannitol with the gellan gum gel. The generated freeze-dried gel network was evaluated in terms of porosity, pore size and wall thickness distributions. The solute physical state was correlated the water activity trend as a function of the solute content. Since mannitol is crystalline, the water activity decreases, in contrast with the amorphous sucrose. The rehydration mechanism was investigated and associated with the solute release from the structure. Specifically, the material properties (surface and bulk) as well as the role of the dissolution medium over time were assessed. It was found that the rehydration for both the gellan/sucrose and gellan/mannitol systems was highly influenced by the additive content, as an increase in water uptake was measured up to 10 wt%. A further increase in solute led to a considerable drop in the rehydration rate and extent due to the change in the freeze-dried structure, with smaller pores and with higher wall thickness values.

**Electronic supplementary material:**

The online version of this article (10.1007/s11483-018-9536-5) contains supplementary material, which is available to authorized users.

## Introduction

Hydrocolloids are of importance in the food industry, as gelling agents, stabilisers or thickeners [1, 2]. They are often used in complex products, such as dairy or instant food, to modulate and enhance their mouthfeel and textural properties [2]. As the quality of the food product is the result of the interaction between the formulation constituents [3], hydrocolloids might behave differently depending on how they interact with other ingredients. Particularly in the confectionery industry, gelling agents are often used in combination with sugars/sweeteners [4].

To improve both preservation and storage, food frequently needs to be dehydrated [5] or to have a reduced moisture content and water activity, depending on the specific industrial applications [6]. To achieve a high product quality, all the formulation constituents and their interaction should be considered during the drying process. Among the common drying techniques, Scherer [7] recommended freeze drying as a method to enhance both product shape and volume preservation, decreasing shrinkage, since it is based on the sublimation of water from the solid matrix, and providing a high-quality structure [8].

Before consumption, dried food is often rehydrated [9]. The water uptake is also dependent on both the structure and properties of the freeze-dried gel embedded within the product formulation, affecting some mechanisms, such as sugar release from the gel network [10].

LA (low-acyl) gellan gum is a microbial polysaccharide [1, 11], extensively used in the food industry [11]. The resulting gel network is highly dependent on the solvent quality [2], affecting the material mechanics [12]. Sugars can promote gellan gum chain aggregation by replacing the solvent [11, 12] and the effectiveness of this behaviour is strictly dependent on the sugar type [2, 13]. However, high sugar contents lead to the inhibition of gellan gum hydration [14] and an excessive biopolymer aggregation, resulting in a sharp decrease in mechanical and textural properties [11]. The reduction in the number of effective junction zones by increasing the solute content leads ultimately to the gellan precipitation [11]. However, the “optimum extent” of association and cross-linking is significantly dependant not only on the sugar content, but also on the presence of salt, which can be contained in the gellan formulation [11].

Mannitol is a sugar alcohol produced by several organisms, commonly used in the food industry as an alternative sweetener [15]. Although mannitol and polyols are referred to as sugar alcohols, they are not actual carbohydrates, as they contain two more hydrogens in their formula due to hydrogenation [15]. It forms a crystalline matrix, which crystal phase depends on the process [16].

After freeze drying, sucrose forms an amorphous matrix from the aqueous solution [17–19]. Since sucrose in its amorphous state is thermodynamically unstable, it may recrystallise over long periods [18, 19], depending on the formulation [17].

Although Huang, Kohashi, Vangundy and Murashige [20] showed the effect of mannitol addition on the mechanical properties of gellan gum, the literature to date does not emphasise the effect of alternative sweeteners, such as mannitol, on the gellan gum gel systems at the molecular level. The different chemical structure between sugars and polyols might affect the gellan gum junction zones and, therefore, the overall network. Whilst Morris, Nishinari and Rinaudo [11] reviewed the role of sucrose in gellan gum gels, there is a lack of information about dried gellan gum/sugar gels. Abramovič and Klofutar [21] reported different models for the drying kinetics, but without referring to freeze drying and just focusing on pure gellan gum gels. Nussinovitch, Corradini, Normand and Peleg [22] investigated the effect of sucrose in freeze-dried gellan, agar and k-carrageenan gels on both the mechanical and acoustic properties, yet the influence of sucrose on the freeze-dried structure, in terms of porosity and pore-wall thickness distribution, was not mentioned.

In this work, the interaction between sucrose and mannitol with low acyl (LA) gellan gum, used as a model gelling agent, was investigated. Firstly, the effect of sugar addition on the molecular gellan gum level was assessed by mDSC and FTIR spectroscopy, as well as the study of the mechanical properties. Sugar content up to 20 wt% were examined. Secondly, the freeze-dried microstructure was evaluated by SEM and μCT microscopy. In particular, the effect of the physical state of mannitol and sucrose on the water activity was considered. Finally, the freeze-dried system was studied in terms of rehydration and leaching mechanisms.

## Materials and Methods

### Gel Preparation

Low acyl gellan gum (Kelcogel F, CPKelco, Surrey, UK) was used as a model gelling agent. The formulation of the CP Kelco gellan gum contains both monovalent and divalent ions (Na^+^, K^+^, Mg^++^, Ca^++^) at around 5.0 *w*/w % [23], mainly added as chlorides [14]. Sugars were added in different mass fractions (5, 10, 15 and 20 wt%). D-Mannitol (>98%, Sigma-Aldrich, Gillingham, UK) and sucrose (>99%, Sigma-Aldrich, Gillingham, UK) were added to hot distilled water at 85 °C, followed by a slow addition of gellan gum (2 wt%). The hot solutions were stirred for two hours at constant temperature for homogeneous mixing.

The gel samples were moulded with a 22 mm diameter and 15 mm height, covered with a plastic film to avoid water evaporation and stored at room temperature (20 °C ± 1 °C) for 24 h before characterisation.

### Molecular Interactions: mDSC and FTIR

Experiments were performed in triplicate from 5 °C to 80 °C with a scan rate of 1 °C/min by using a micro DSC 3 evo (Seteram Instrumentation, Caluire, France). The gel sample was placed in a screw-top cell, using distilled water in the reference cell. A series of two heating/cooling cycles was applied with isothermal periods to reduce the thermal history effect.

Molecular interactions between the gellan gum and sugars were evaluated by FTIR Spectroscopy (Spectrum Two IR Spectrometer, Perkin Elmer, Waltham, MA, USA) in reflection mode within the wave numbers range 600–4000 cm^−1^. For each sample 16 scans were applied with a resolution of 4 cm^−1^.

### Texture Analysis

Mechanical properties were evaluated in triplicate by using the texture analyser TA.XT.plus (Stable Micro Systems Ltd., Godalming, UK) with a 40-mm-diameter cylindrical aluminium probe fitted. Both the Young’s modulus and the gel strength generated by a strain compression of 50% were measured. A thin layer of silicone oil was applied on the probe plates to reduce the friction during the compression, performed with 2 mm/s rate. All the measurements were carried out in triplicate for the statistical analysis. The gel compression was plotted in force/distance, reporting a plus/minus standard deviation on the curve every 0.5 mm.

### Freeze Drying

The gel samples were put into a − 18 °C freezer for 24 h, applying a slow freezing rate of around 0.2 °C/min. Afterwards, they were placed into the freeze dryer (SCANVAC 110–4 PRO, LaboGene, Lillerød, Denmark) onto the shelf trays for 48 h. The condenser temperature was set at −110 °C and the working pressure in the chamber was decreased to 0.18 mbar by a rotary pump, below the triple point of water. These process conditions were defined by the equipment and were kept constant for all the experiments.

After drying, the samples were stored under low-vacuum conditions in a desiccator until characterisation, performed within 12 h from the end of the freeze drying.

### Normalised Moisture Content (NMC) and Water Activity

*NMC* (Normalised Moisture Content) [5] was used to monitor water content in triplicate at the end of the drying process and during rehydration (Eq. 1).

1 $$ NMC=\frac{\frac{\left( Md- Ms\right)}{Ms}}{\frac{\left( Mo- Ms\right)}{Ms}}=\frac{\left( Md- Ms\right)}{\left( Mo- Ms\right)} $$

Where *M*_*d*_ is the sample mass after drying (or during rehydration), *M*_*s*_ the solid sample mass, and *M*_*o*_ the pre-dried sample. *M*_*o*_ was measured before putting the gels into the freezer.

The negligible moisture content threshold is suggested by Brown [24] as *NMC* < 0.1.

The Aqualab dew point water activity meter 4te (Labcell LTD, Alton, UK) was used to measure the water activity values. The gel samples were placed into the test chamber at 25 °C, after being crushed to analyse a_w_ throughout the sample.

### Micro Computed Tomography (μCT) and Scanning Electron Microscopy

Micro computed tomography (Bruker microCT, SkyScan 1172, Evere, Belgium) was performed to quantitatively analyse the total porosity. This system allows visualisation of 2D cross-sections and generates a complete 3D structure reconstruction without any chemical fixation. The acquisition mode can be set at a maximum current of 96 μA and voltage of 100 kV. Qualitative and quantitative analyses were performed using a CT-analyser (1.7.0.0), after binarisation into black and white images, obtaining porosity information. The “sphere-fitting” algorithm was applied for the structure separation and thickness calculations.

ESEM FEG (XL30, Philips, Amsterdam, The Netherlands) was used to collect high-quality micrographs of the dried gel/sugar structures. After cooling in liquid nitrogen, the dried gels were cut in both vertical and horizontal direction. The maximum voltage was set up to 10 kV and the magnification to × 150.

### Rehydration and Leaching

The water uptake expressed as *NMC* (Eq. 1) was evaluated by measuring the samples weight in triplicate every 3 min for 30 min. The gel samples, completely submerged in distilled water (100 ml) at room temperature (20 °C ± 1 °C), were gently blotted before weighing. The *NMC* values were adjusted taking into consideration the solid evolution over time, due to the sugar release into water. In particular, M_s_ was re-calculated at each time: the amount of sugar in solution was measured and subtracted from the initial solid content in the sample.

Sugar release was evalueted by using a small-volume, temperature-controlled, automatic refractometer (J357, Rudolph Reasearch Analytical, Hackettstown, NJ, USA). The dried samples were located in 100 ml of distilled water at room temperature (20 °C ± 1 °C), simulating sink conditions [25], as the the saturation volume was more than 5–10 times smaller than the volume of the medium. The measurement was carried out on 0.5 ml of solution, withdrawn every 3 min up to 30 min. The analysed solution was pipetted into the original solution to avoid increasing the concentration as an artefact. The plotted graphs present normalised curves expressed as Release Ratio (*RT*) on a scale from 0 to 1 (Eq. 2).

2 $$ Release\ Ratio\ (RT)=\frac{\frac{g\  sugar}{g\  water}(t)}{\frac{g\  sugar\ \mathit{\max}}{g\  water}} $$

Where g sugar/g water is the experimental value as a function of time, while g sugar max/g water represents the concentration of the the amount of sugar constituting the dried sample.

The Higuchi model (Eq. 3) was used to evaluate the solute release from the dried gel structure in terms of quality of fit by R^2^ and compared with a linear fitting [26].

3 $$ {f}_t={K}_H{t}^{0.5} $$

Where *f*_*t*_ is the amount of released sugar into the solution by surface unity and is *K*_*H*_ stands for the Higuchi dissulution constant.

Costa and Lobo [26] suggested that the Higuchi model is particularly suitable for modelling the release of active compounds from porous materials, since it takes into consideration the structure parameters of the dried gel system, such as the porosity [26], as reported in Eq. 4.

4 $$ {f}_t=\sqrt{D_B{C}_st\left[2{\rho}_d{\upvarepsilon}_d^a-\left({\upvarepsilon}_i+{\upvarepsilon}_d^a\ \right){C}_s\right]} $$

Where *D*_*B*_ [m^2^/min] is the diffusion coefficient through the matrix channels, *C*_*s*_ and *ρ*_*d*_ are respectively the solubility [g/m^3^] in the matrix/aqueous solution and the solid-state density of sugar [g/m^3^]. ${\varepsilon}_d^a$is the accesible drug porosity (i.e. the volume fraction of the loaded solute that can be solubilised by the dissolution medium), ɛ_*i*_ the inherent porosity (i.e. initial porosity, before dissolution).

### Wettability and Static Contact Angle

Wettability was assessd in triplicate by measuring the static contact angle at room temperature (20 °C ± 1 °C) by using the KRÜSS Drop Shape Analyser (DSA 100, Hamburg, Germany). The dried samples were compacted into circular tablets (1 cm in diameter and 3 mm in height) to obtain a flat surface by a hydraulic press. A 500 μL glass syringe with a 0.5 mm needle diameter was used to deposit a 5 μl distilled water drop onto the dried material. The measurement was carried out in triplicate using the value at 1 s after droplet deposition.

## Results and Discussion

### Pre-Dried Gels

**Fig. 1:**
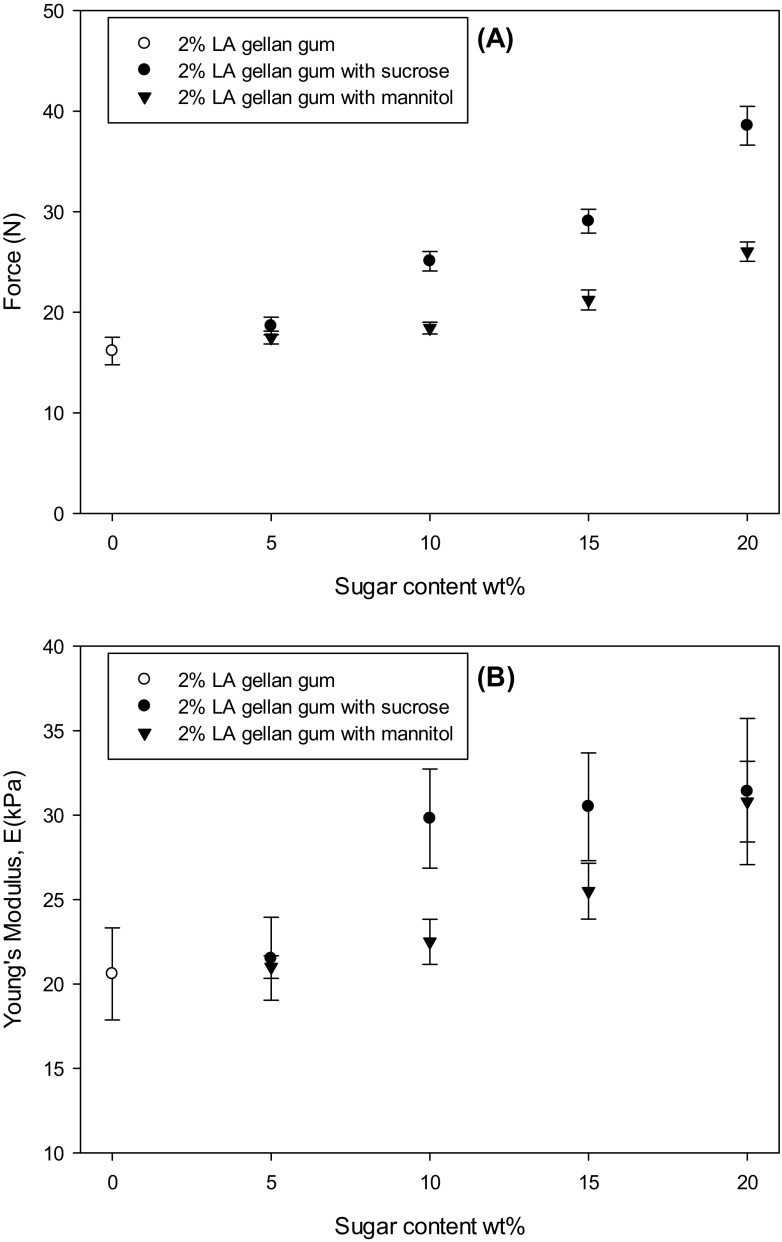
Gel strength **a** and Young’s modulus **b** as a function of sugar type (● sucrose, ▼ mannitol) and content. Gellan gum without sugars (○)

Fig. 1a-b show the mechanical properties of the gels as a function of the sugar type and content. The peak force generated by a 50% strain compression increased with the sugar content, especially from 10 wt% up, whereas only a slight increase was observed for Young’s modulus.

The gel strength increases as the solvent is reduced by replacement with solid content. This leads to a higher polymer chain aggregation. The slight elastic modulus increase may suggest that the number of effective junction zones does not increase, although the polymer is more aggregated. Instead, the gellan gum network is likely to be only morphologically entangled.

In Fig. 1a-b it is possible to observe that the mechanical responses are different, depending on the sugar type. Specifically, mannitol shows less resistance to compression, as the gel strength is lower than the gellan/sucrose system (Fig. 1a). This might be related to an improved network lubrication compared to sucrose [27]. Depending on the size of the sugar molecule, the polymer chains can move differently [28]. Since the molecular weight of mannitol is 182.2 g mol^−1^, lower than sucrose (342.3 g mol^−1^), it might penetrate more the interstitial parts of the gel network, lubricating it. However, the the extent in gel network lubrication is dependent also on other parameters related to the co-solute, such as hydrogen bonding, solubility, polarity and dialectric constant [28].

**Fig. 2:**
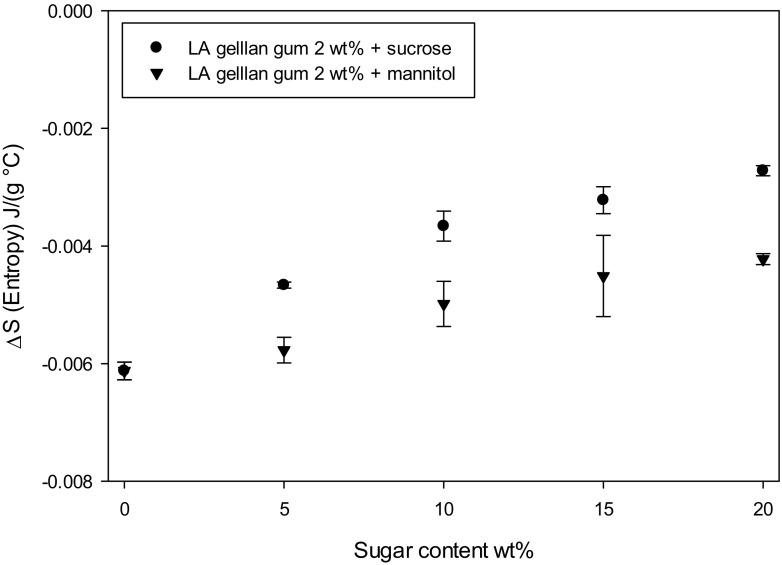
Entropy difference (*ΔS*) as a function of sugar type (● sucrose, ▼ mannitol) and content. Data are referred on cooling

The molecular structure was further investigated with mDSC to correlate the gel structure order/aggregation with the mechanical properties. It was found that the gel network order decreases as sugar increases, since there is a less pronounced reduction in entropy *ΔS* on cooling, compared to the value for 0 wt% sugar (Fig. 2). This thermal transition is referred to the gellan gum coil-helix and sol-gel transitions [29]. *ΔS* is estimated as *ΔH/T*_*p*_, where *T*_*p*_ stands for the peak temperature, balancing the enthalpic interactions and the entropic value at the equilibrium (supplementary Table S1), where *ΔG = 0 = ΔH-T*_*p*_*ΔS* [12].

Although there are more interactions between the polymer chains and between the polymer and sugar due to the solvent reduction by solute replacement and a more packed structure is formed, these chains are more disordered. Specifically, mannitol less affects the structure order, since the the entropy remains closer to the system at 0 wt% sugar (Fig. 2). It might be related to the smaller molecular size for mannitol, which could less affect the network order.

**Fig. 3:**
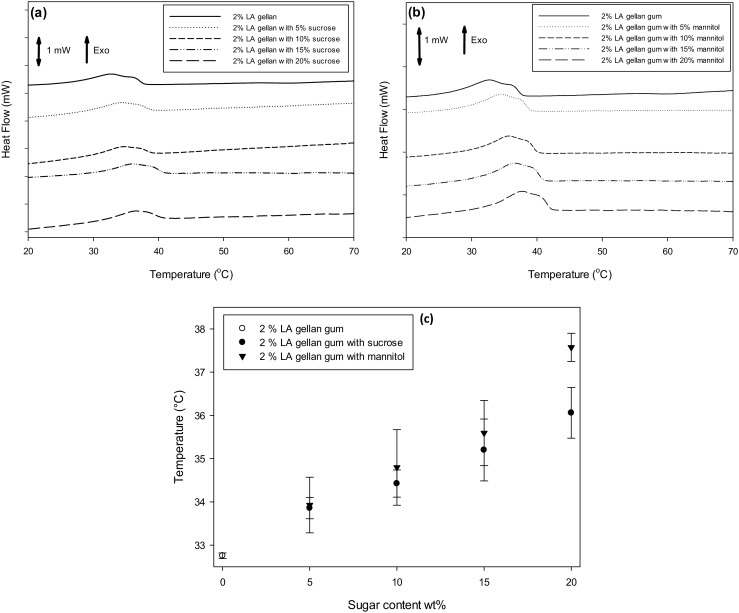
mDSC curves on cooling for gellan/sucrose **a** and gellan/mannitol **b**. Peak temperature as a function of sugar type **c** (● sucrose, ▼ mannitol) and content. Gellan gum without sugars (○)

As previously mentioned, the water reduction leads to a more aggregated structure, confirmed by the transition peak temperature increase by adding both sucrose and mannitol (Fig. 3a-b-c).

**Fig. 4:**
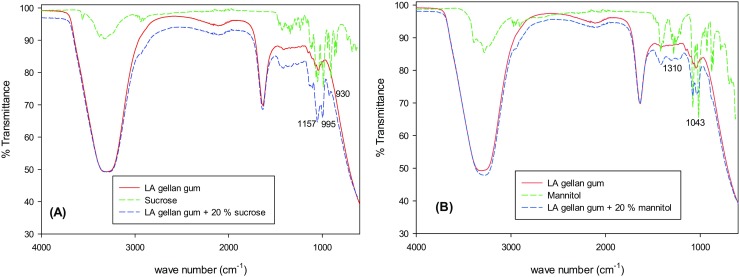
FTIR spectra for gellan/sucrose **a** and gellan/mannitol **b**. Red line is related to LA gellan gum gel, green to pure sucrose or mannitol, blue to LA gellan gum +20 wt% sugar. The peak wave numbers are referred to gellan/sucrose and gellan/mannitol

The molecular interaction of the sugars with the gel polymer is confirmed by the FTIR (Fig. 4a-b). In fact, the characteristic sucrose bands are shifted from 1145, 980 and 903 cm^−1^ to 1157, 995 and 930 cm^−1^ respectively. Noor, Majid, Arof, Djurado, Neto and Pawlicka [30] reported similar considerations in gellan gum-LiCF_3_SO_3_. However, the formation of new complex was not observed, since there were no different peaks in the mixed system.

Mannitol generates similar interactions, since the peaks at 1278 and 1014 cm^−1^ are shifted to higher wave numbers, specifically 1310 and 1043 cm^−1^.

### Freeze-Dried Gels

The freeze-drying process was performed for 48 h as a reference time, since it was found that for all the samples with either mannitol or sucrose up to 20 wt%, the *NMC* value is below 0.1, set as a reference for negligible moisture content [5].

The water activity was measured to first investigate the solute physical state (i.e. crystalline or amorphous solid) and correlate it with the mechanical and structural properties. The water activity values for the 48-h freeze-dried gellan gel with both sucrose and mannitol are shown in supplementary Fig. S1.

As the mannitol content increases, a_w_ decreases, whereas the trend for sucrose is completely different. This behaviour is related to the solute physical state [6, 19]. In particular, Yu, Milton, Groleau, Mishra and Vansickle [16] reported that hydrated crystalline materials, as freeze-dried mannitol, have structural water, difficult to remove during the drying process. The water molecules form hydrogen bonds and are localised in the crystal lattice [31]. Therefore, by increasing the mannitol content, the percentage of crystalline material rises and, as a result, the overall amount of free water within the product is expected to decrease. On the other hand, the amount of water in amorphous substances, such as sucrose in freeze-dried products, can substantially be higher [32], acting as a plasticiser [33]. Water can enhance the time-dependent recrystallization process [6], leading to the water desorption from the material [33, 34] and to the increase of apparent water activity, if moisture is not removed from the product [32]. Therefore, since water activity can change as a function of time during storage [6, 34], all the dried gels are measured within a few hours after the end of the freeze-drying process.

**Fig. 5:**
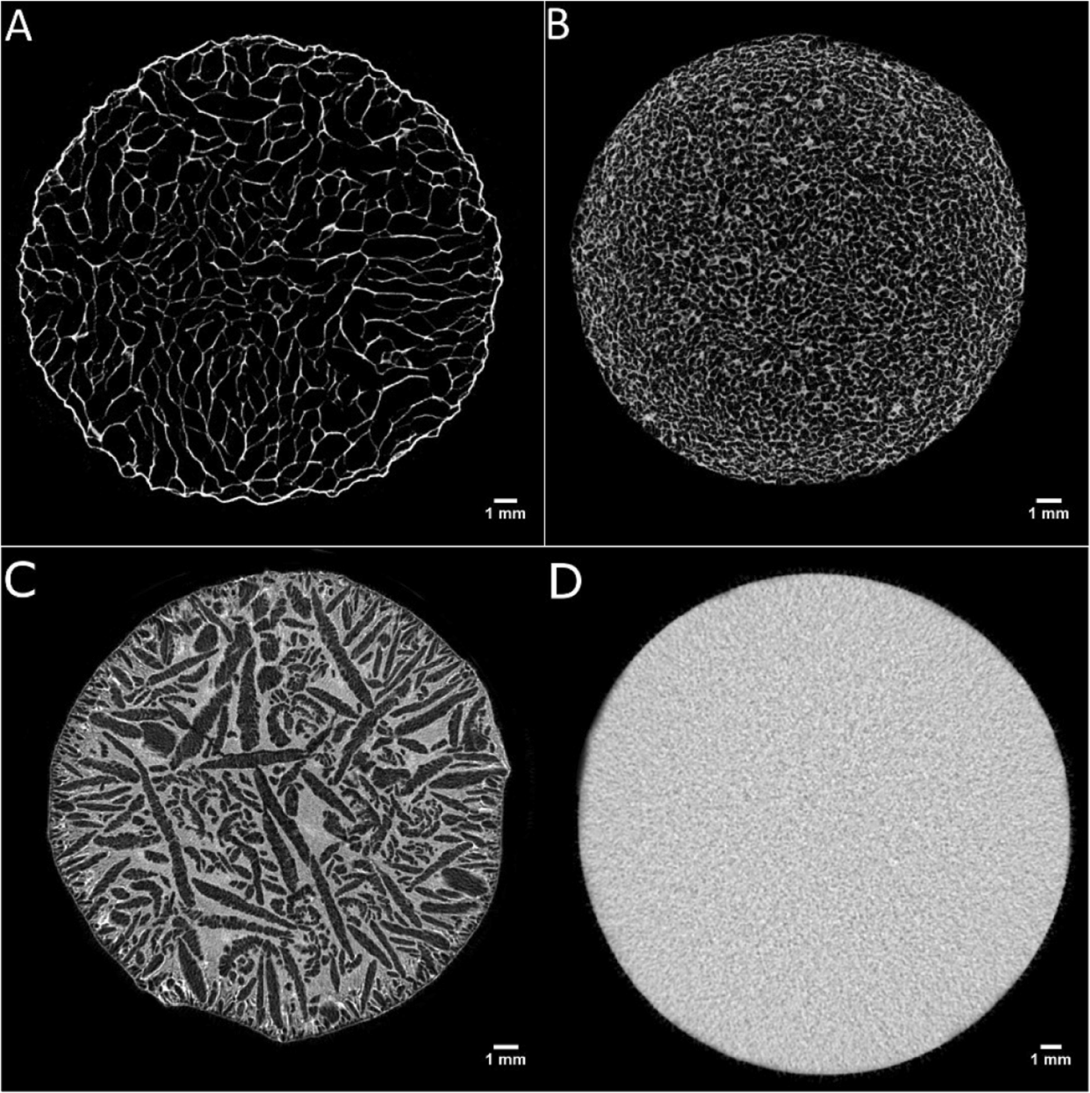
μCT, freeze-dried microstructures: 2 wt% LA gellan gum **a**, 2 wt% LA gellan gum +20 wt% sucrose **b**, 2 wt% LA gellan gum +20 wt% mannitol **c**. Pre-dried sample, 2 wt% LA gellan gum (D, reference)

The freeze-dried microstructure was initially analysed by μCT (Fig. 5a-b-c-d). The total porosity for the dried gel without sugar was 84.8 ± 4.2%. The sucrose addition at 10 wt% and 20 wt% led to a drop to 73.9 ± 0.5% and 48.6 ± 2.9% respectively, while the corresponding values for mannitol/gellan system were 64.5 ± 0.4% (10 wt%) and 50.1 ± 1.8% (20 wt%).

**Fig. 6:**
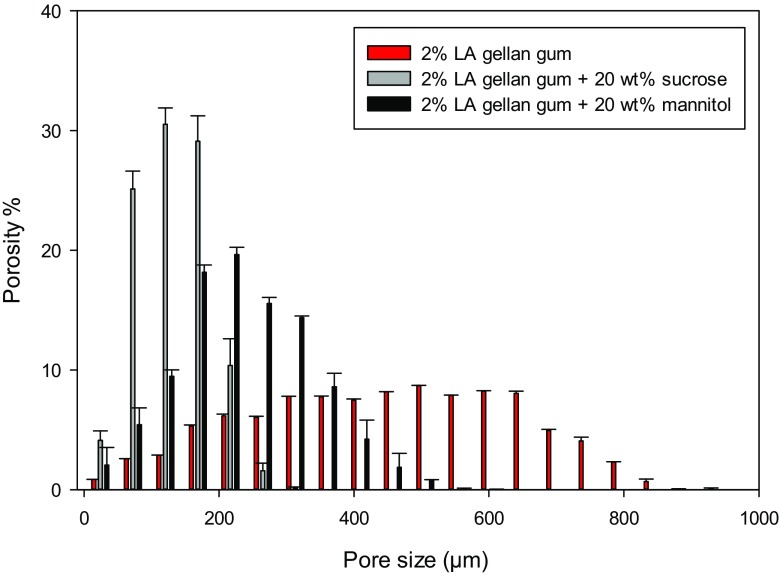
Pore size distribution for freeze-dried LA gellan gum +20 wt% sugar (grey-sucrose, black-mannitol). Freeze-dried LA gellan gum is in red

Overall, the presence of sugar completely affected the dried structure, decreasing the final porosity and shifting the pore average size towards smaller values (Fig. 6).

From the pore distribution, mannitol generated slightly larger pores compared to sucrose. As the structure of freeze-dried materials is dependent on the freezing step, the difference between gellan/mannitol and gellan/sucrose may be related to a different supercooling [35], since it is well-known that sugar alcohol compounds depress more the freezing point than sucrose and other disaccharides [36]. Consequently, a higher degree of supercooling might be expected for sucrose, enhancing the ice crystal nucleation and limiting the crystal growth.

**Fig. 7:**
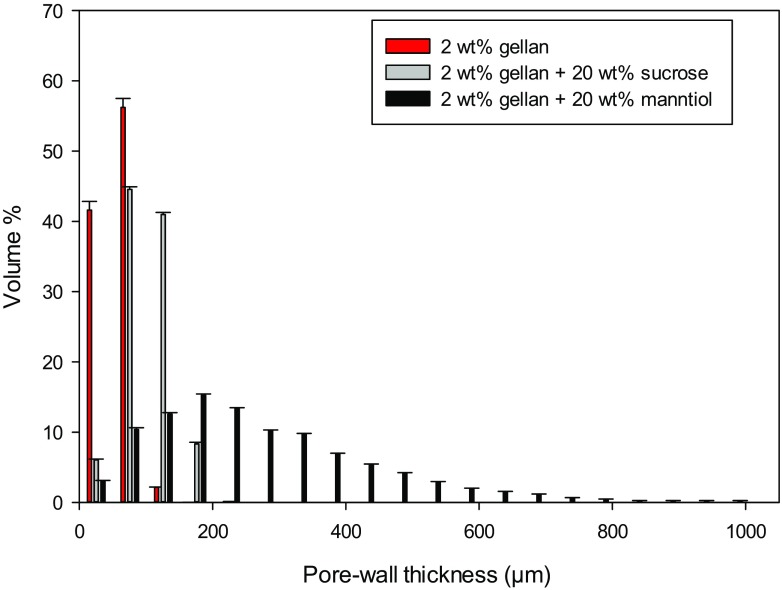
Pore-wall thickness distribution for freeze-dried LA gellan gum +20 wt% sugar (grey-sucrose, black-mannitol). Freeze-dried LA gellan gum is in red

The main difference between the two systems is that sucrose and mannitol lead to different porosity characteristics. Specifically, the former produced small and circular voids within the cross-section. The latter generated large and elongated pore clusters along a direction. In effect, the micrographs in Fig. 5 suggest that the pore-wall thickness is considerably different, as confirmed in Fig. 7.

Mannitol generated pore clusters more separated by solid walls, affecting the pore interconnection. On the other hand, the pore-wall thickness formed by sucrose was more similar to the freeze-dried gellan without additives. Interestingly, both the pore and wall thickness distributions were more comparable between the two systems if the sugar content is 10 wt%. These results are likely to be related to the mannitol solubility in water, as mannitol can be precipitated during both the gel cooling and the freezing step, especially at content above 10 wt%. In effect, the mannitol solubility in water at around 10 °C is ~13 wt% [37], while at 80 °C it is above 45 wt% [15, 37]. The mannitol precipitation might also lead to higher values of pore-wall thickness as well as a specific direction of the pore clusters, as solid material may interfere with the ice crystal growth.

**Fig. 8:**
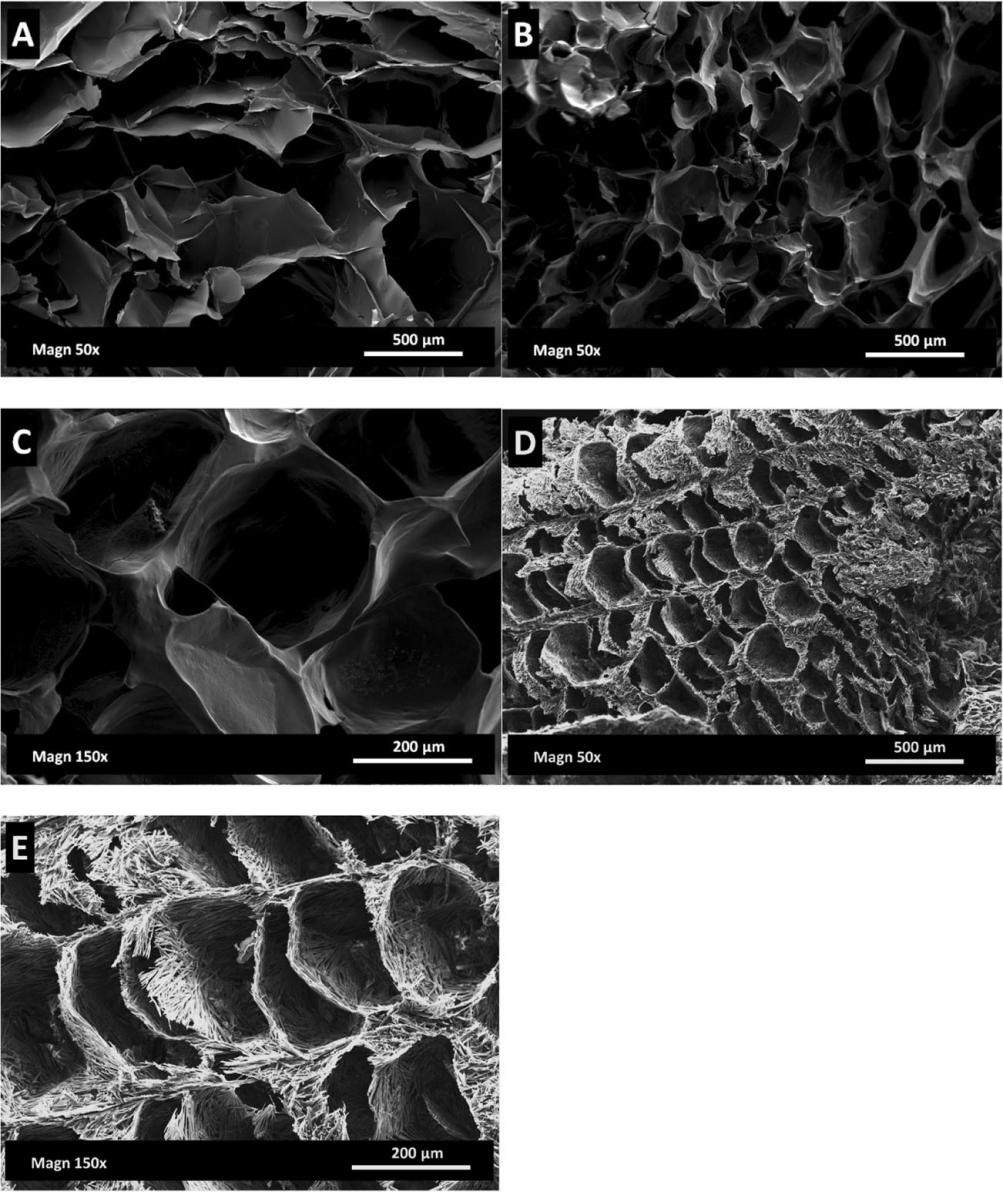
ESEM, freeze-dried microstructures: 2 wt% LA gellan gum **a**, 2 wt% LA gellan gum +20 wt% sucrose **b**, **c**, 2 wt% LA gellan gum +20 wt% mannitol **d**, **e**

In Fig. 8, the ESEM results provide more information about the dried material. The gellan/sucrose dried gel presents a homogeneous matrix (Fig. 8b-c). Devi and Williams [38] reported SEM micrographs for a freeze-dried sucrose solution at 5% *w*/w with similar solid dried walls, suggesting that sucrose forms an amorphous phase. On the other hand, mannitol forms crystals around the pores (Fig. 8d-e). The shape of these mannitol crystals after a freeze-drying process is in agreement with the current literature [39].

Interestingly, it is observed that sucrose does not produce the collapse of the freeze-dried structure, although the drying process is carried out at a temperature higher than the collapse temperature for pure sucrose (Tc = ~ − 32 °C) [40]. In effect, Fig. 8b-c show a homogeneous structure, without domains with different density in the solid material, as Rey and May [41] reported for the system glucose/mannitol, or with a small scale collapse [18]. It may indicate that the interaction gellan-sucrose has a positive effect, preventing the structure collapse during freeze drying.

**Fig. 9:**
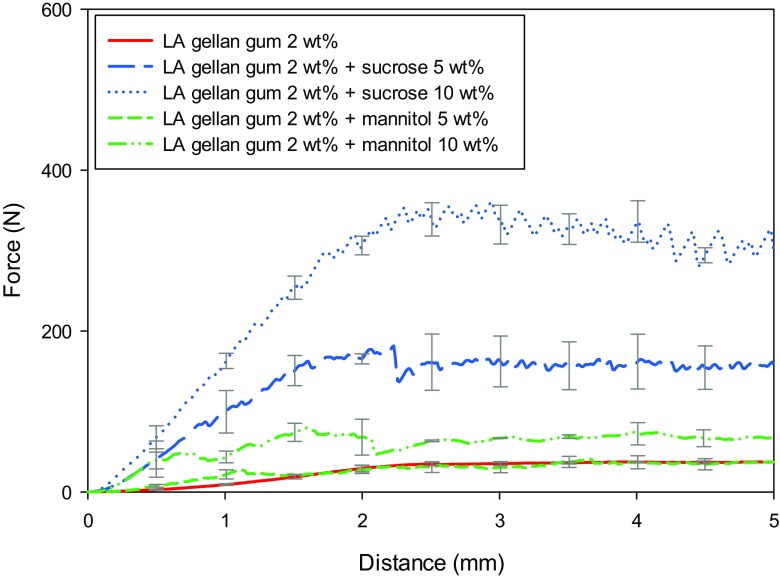
Force (N) vs distance (mm) for gellan/sucrose (blue) and gellan/mannitol (green). Red solid line refers to the dried gel without sugars

These dried structures generated completely different mechanical properties as shown in Fig. 9, in agreement with Devi and Williams [38]. It is noticed that the deformation mechanism occurs through a succession of abrupt fractures during compression [38]. After the initial linear elastic behaviour, a sharp drop in the applied force follows each brittle cracking, especially evident for the gellan-sucrose system.

It seems that the mechanical properties of the dried material depend on both the formulation, as noticed for the pre-dried samples, and the different microstructure, namely porosity, void location and wall thickness [42]. Moreover, the particular physical state of sugar might contribute to the overall mechanical behaviour. In this context, the SEM micrographs may suggest that a structure formed by more fibre-like crystals, as the case of mannitol/gellan, can be deformed more easily under an applied stress, caused by the slide between these crystals.

### Rehydration and Leaching

**Fig. 10:**
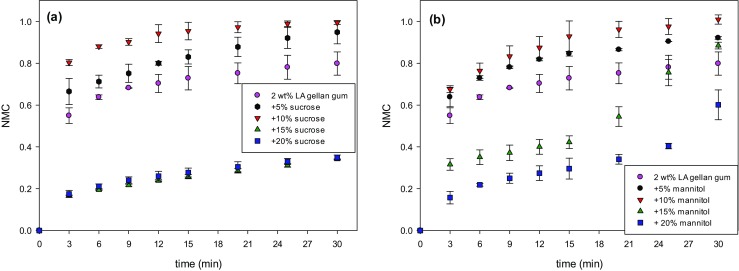
Rehydration expressed as *NMC* over time as a function of sucrose **a** or mannitol **b** and content

Fig. 10a-b show the rehydration curves for gellan-sucrose and gellan-mannitol.

Sucrose presented a slightly quicker water uptake over time compared to mannitol, especially at sugar contents up to 10 wt%. However, at 20 wt%, this difference was negligible. In both cases, there was an increase in rehydration rate and extent as a function of sugar amount. However, at a content equal or above 10 wt%, the water uptake kinetics sharply dropped.

To have a better understanding of the rehydration mechanism, both the surface and bulk properties of the material need to be considered, as well as the medium properties as sugar starts to dissolve in water [43].

The surface properties were assessed in terms of material wettability by measuring the static contact angle. As the sugar content increases, the average wettability becomes higher (Table 1) and, consequently, the rehydration rate is expected to rise as sugar increases.

**Table 1:** Contact angle as a function of sucrose and mannitol contents. LA gellan gum is kept at 2 wt%

		Contact angle [°]
LA gellan gum 2%		78.5 ± 1.2
+ Sucrose (wt%)	5	60.4 ± 0.6
	10	47.5 ± 2.4
	20	28.0 ± 3.3
+ Mannitol (wt%)	5	62.4. ± 2.3
	10	52.7 ± 3.8
	20	11.5 ± 3.1

In terms of bulk properties, the total porosity and pore distribution were not significantly different between gellan gum with sucrose or mannitol, although the average pore volume was slightly lower for the latter. A possible reason for sucrose/gellan to rehydrate slightly more quickly over time especially at a low solute content can lie in the pore-wall thickness, since it is found to be considerably thinner for sucrose/gellan (Fig. 7). Water can penetrate into the structure more easily, passing through a thinner wall that separates two non-connected pores. As a result, this offers less resistance to the water absorption. The decrease in rehydration rate for both systems, above 10 wt%, may be due to an enhanced structure packing, with thicker walls.

**Fig. 11:**
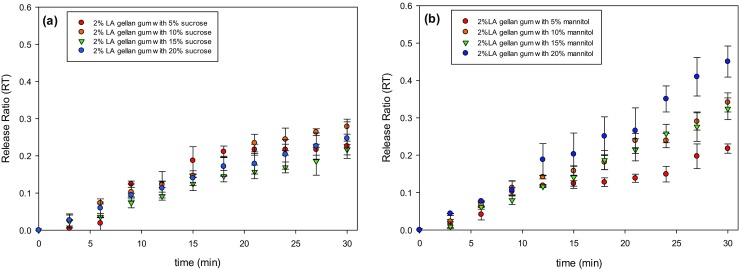
Sugar release over time as a function of sucrose **a** or mannitol **b** and content

Finally, the medium properties evolve over time, as the viscosity increased as sugar was released. The viscosity values of the aqueous solution with either sucrose or mannitol were similar at a given concentration, within the employed range of solute contents [44, 45]. After the initial sugar dissolution and release at the interface between the sample and the solution, both mannitol and sucrose should diffuse through the rehydrated structure into the aqueous solution by the gradient of concentration. The normalised sugar release curves are shown in Fig. 11a-b. It seems that the dissolution rate for mannitol (Fig. 11b) was slightly higher compared to sucrose (Fig. 11a). Interestingly, after 30 min the amount of sugar in solution was not equal to the initial solute mass of the dried sample, as the release value did not reach 1. It suggests that the sugar in the core of the sample required more time to diffuse out. In effect, since the rehydrated mannitol/gellan structure became visually less compacted over time, due to the solid leaching, and more solute was released from the inner parts of the gel, a change in slope at longer time scales was observed (Fig. 11b). That can also explain why the error bars related to mannitol/gellan are larger than the system with sucrose.

To support this theory, the gels with both the solutes were squashed to encourage a complete solute dissolution. In this case, the release ratio was close to 1.

In supplementary Fig. S2 the dissolution process for both 10 wt% gellan/sucrose and gellan/mannitol was fitted by Higuchi model (Eq. 4), adapted for a porous matrix [26]. It has been suggested that this model, based on the Fickian diffusion in a square-root time dependence, is appropriate to describe release phenomena [26].

However, it seems that the Higuchi model overestimates the material release with respect to the experimental points, especially for short timescales. It may be due to the assumptions made to model the system. Specifically, *D*_*B*_ is considered to be equal to the diffusion coefficient in water at 20 °C, without any adjustment to represent the diffusion within the interconnected pores of the matrix [46]. In addition, the system is actually not planar and the sample shape slightly evolves over time, due to the effect of swelling.

By contrast, a linear fitting is more accurate, with the R^2^ of 0.983 and 0.987 respectively for the gellan/sucrose and gellan/mannitol systems. This relatively slow release is likely to be due to the progressively thicker external layer of the sample that forms over time.

From the previous considerations, both the material and medium properties are likely to affect the rehydration mechanism. A larger sugar amount should enhance the wettability, yet make the structure more packed, with thicker walls and less pores.

It is noteworthy to mention that the initial gel properties cannot be recovered, offering no-resistance to compression. Although the formation of ice crystals during the freezing step align and aggregate the polymer gellan gum chains along the ice crystal edges, the freeze-dried process generates large pores that are cracks within the material. Moreover, during rehydration the solute release occurs, leaving a less compacted material with a lower solid content.

## Conclusions

In the present work, results on freeze-dried low-acyl gellan gum gels with sucrose or mannitol are for the first time reported. The freeze-dried microstructures were studied as well as the rehydration and solute-release mechanisms. Before drying, the presence of either sucrose or mannitol leads to the gel aggregation, due to the solvent replacement with solute, and, at the same time, a less ordered gel network. This gel structure aggregation produces stronger gels, as the polymer chains are closer and more entangled. Specifically, the gellan/sucrose system shows higher values of gel strength, probably due to the different molecular steric hindrance and gel network lubrication compared to mannitol. The generated gel structure after freeze drying has a considerable effect on the mechanical properties, as the specific additive type generates a different porosity as well as pore/pore-wall thickness distributions. Specifically, mannitol generates thicker walls around the pores, whereas sucrose leads to smaller pores and thinner walls. These structural parameters affected the rehydration rate/extent as well as the solute release from the dried gel network. Both gellan/mannitol and gellan/sucrose systems showed an initial increase in rehydration rate, followed by a considerable drop to higher solute contents.

## Electronic supplementary material

ESM 1 (DOCX 85 kb)
