# Supplementary material for: Interaction of Mannitol and Sucrose with Gellan Gum in Freeze-Dried Gel Systems
Source: Food Biophys. 2018 May 22;13(3):304–15. doi: 10.1007/s11483-018-9536-5 (PMC6061513; doi:10.1007/s11483-018-9536-5)
Supplement: Supplementary file 1 — (DOCX 85 kb) [file 11483_2018_9536_MOESM1_ESM.docx]

**SUPPLEMENTARY DATA**

**Interaction of mannitol and sucrose with gellan gum in freeze-dried gel systems**

Mattia Cassanelli^1*^, Ian Norton^1^ and Tom Mills^1^

^1^School of Chemical Engineering, University of Birmingham, Edgbaston, Birmingham, B15 2TT, UK

*mxc446@student.bham.ac.uk

**Fig. S1**

Water activity (a_w_) as a function of sugar type (● sucrose, ⯆mannitol) and content. Gellan gum without sugars (○).


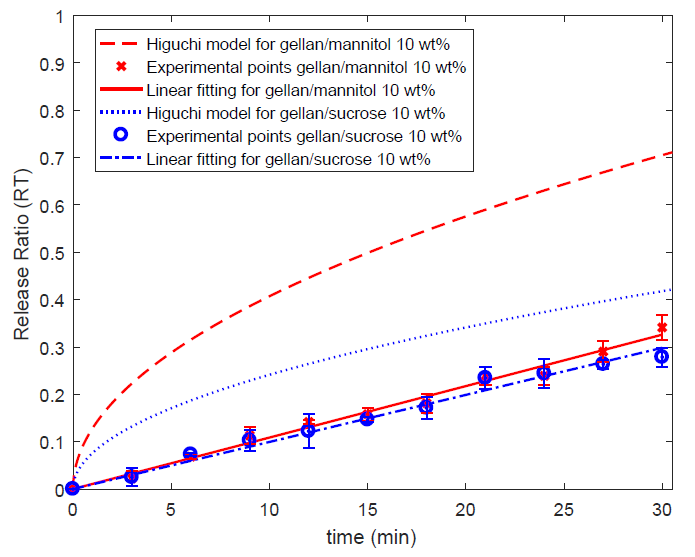


**Fig. S2**

Dissolution process: Higuchi model (eq. 4) for both 10 wt% gellan/sucrose and gellan/mannitol.

**Table S1**

Peak temperatures, enthalpies and entropies measured on cooling as a function of mannitol or sucrose content.

|  | T_p_ (°C) | ΔH (J g^-1^) | ΔS (J g^-1^ °C ^-1^) * 10^-3^ |
| --- | --- | --- | --- |
| Gellan gum 2 wt% | 32.7 ± 0.1 | -0.200 ± 0.005 | -6.1 ± 0.1 |
| + Sucrose 5 wt% | 33.8 ± 0.2 | -0.158 ± 0.03 | -4.6 ± 0.1 |
| + Sucrose 10 wt% | \| 34.4 ± 0.3 \| \| --- \| | -0.126 ± 0.008 | -3.6 ± 0.2 |
| + Sucrose 15 wt% | \| 35.2 ± 0.7 \| \| --- \| | -0.113 ± 0.008 | -3.2 ± 0.2 |
| + Sucrose 20 wt% | \| 36.1 ± 0.6 \| \| --- \| | -0.098 ± 0.002 | -2.7 ± 0.1 |
| + Mannitol 5 wt% | \| 33.9 ± 0.6 \| \| --- \| | -0.195 ± 0.003 | -5.7 ± 0.2 |
| + Mannitol 10 wt% | \| 34.8 ± 0.9 \| \| --- \| | -0.173 ± 0.016 | -4.9 ± 0.4 |
| + Mannitol 15 wt% | \| 35.6 ± 0.7 \| \| --- \| | -0.160 ± 0.025 | -4.5 ± 0.7 |
| + Mannitol 20 wt% | \| 37.5 ± 0.3 \| \| --- \| | -0.158 ± 0.004 | -4.2 ± 0.1 |
